# Droplet Digital PCR (ddPCR) Analysis for the Detection and Quantification of Cow DNA in Buffalo Mozzarella Cheese

**DOI:** 10.3390/ani11051270

**Published:** 2021-04-28

**Authors:** Anna Cutarelli, Andrea Fulgione, Pasquale Fraulo, Francesco Paolo Serpe, Pasquale Gallo, Loredana Biondi, Federica Corrado, Angelo Citro, Federico Capuano

**Affiliations:** 1 Department of Food Inspection, Istituto Zooprofilattico Sperimentale del Mezzogiorno, Via Salute 2, 80055 Portici, Italy; andrea.fulgione@izsmportici.it (A.F.); loredana.biondi@izsmportici.it (L.B.); federica.corrado@cert.izsmportici.it (F.C.); federico.capuano@cert.izsmportici.it (F.C.); 2 National Reference Centre for Hygiene and Technologies of Water Buffalo Farming and Productions, Istituto Zooprofilattico Sperimentale del Mezzogiorno, Via delle Calabrie 27, 84132 Salerno, Italy; pasquale.fraulo@cert.izsmportici.it; 3 Department of Chemistry, Istituto Zooprofilattico Sperimentale del Mezzogiorno, Via Salute 2, 80055 Portici, Italy; francescopaolo.serpe@cert.izsmportici.it (F.P.S.); pasquale.gallo@cert.izsmportici.it (P.G.); 4 Veterinary Health Unit of Battipaglia, Azienda Sanitaria Salerno, Via Fiorignano 1, 84091 Battipaglia, Italy; a.citro@aslsalerno.it

**Keywords:** PDO buffalo mozzarella cheese, real-time PCR, ddPCR, isoelectric focusing

## Abstract

**Simple Summary:**

Buffalo mozzarella cheese, sold as a Protected Designation of Origin (PDO) product, is made exclusively from Mediterranean buffalo (*Bubalus bubalis*) milk. To maximize their profits and overcome seasonal shortages of buffalo milk, some producers have started to produce “PDO” buffalo mozzarella cheese by adulterating buffalo milk with milk from different species. such as bovine, thus defrauding consumers. This practice has led the Italian government to reinforce controls on food mislabeling and fraud through traceability mechanisms. The aim of this work is the development of a molecular assay—droplet digital Polymerase Chain Reaction technique (ddPCR)—able to detect the DNA of cow and/or buffalo milk in PDO buffalo mozzarella cheese, thus revealing fraud. The results have highlighted that, thanks to its high precision and sensitivity, the ddPCR could represent an efficacious means of supporting the official controls aimed at combating the adulteration of buffalo mozzarella cheese with bovine milk.

**Abstract:**

Buffalo mozzarella cheese is one of the most appreciated traditional Italian products and it is certified as a Protected Designation of Origin (PDO) product under the European Commission Regulation No. 1151/2012. It is obtained exclusively from buffalo milk. If made from cow milk, or a mixture of buffalo and cow milk, buffalo mozzarella cheese does not qualify as a PDO product. In order to maximize their profits, some producers market buffalo mozzarella that also contains cow milk as a PDO product, thus defrauding consumers. New methods for revealing this fraud are therefore needed. One such method is the droplet digital Polymerase Chain Reaction (ddPCR). Thanks to its high precision and sensitivity, the ddPCR could prove an efficacious means for detecting the presence of cow milk in buffalo mozzarella cheese that is marketed as a PDO product. ddPCR has proved able to detect the DNA of cow and/or buffalo milk in 33 buffalo mozzarella cheeses labelled as PDO products, and experimental evidence could support its application in routine analyses.

## 1. Introduction

Buffalo mozzarella cheese bearing the Protected Designation of Origin (PDO) mark is made exclusively from fresh Mediterranean buffalo (*Bubalus bubalis*) milk, and it is one of the most appreciated Italian foodstuffs.

The fresh whole milk used for the production of PDO buffalo mozzarella cheese must present the following composition: 7.2% of fat and a minimum protein of 4.2%. It also has to be delivered and processed within 60 h of the first milking, as reported by the MIPAAF Decree [1]. Milk with these characteristics is heated until it reaches 63 °C (+2 °C) for 15 s, then cooled to 38–39 °C. Later, the whey starter and rennet are added, thus obtaining the curd. After maturation (pH 4.5–5.0), the curd is heated in water until it reaches 96 °C (+2 °C) and “strings” of mozzarella are formed. Then, the strings are shaped into spheres of different dimensions, cooled in cold water, and salted [2]. 

It has been observed that, in order to maximize their profits and overcome seasonal shortages of buffalo milk, some producers occasionally produce “PDO” buffalo mozzarella cheese by adulterating buffalo milk with milk from different species. This fraud, which is mainly motivated by the fact that buffalo milk costs more than that of other species, may also be encouraged by the difficulties associated with the rheological characteristics of buffalo milk caseins, which make the stretching and mechanical spinning of buffalo mozzarella cheese laborious [3].

In light of the above considerations, food traceability and fraud control play a significant role in guaranteeing the PDO denomination and, consequently, reassuring consumers as to the truthfulness of the product’s label [4]. The most common form of adulteration involves supplementing or replacing buffalo milk with less expensive cow milk. This adulteration negatively influences some nutritional indices of the product; in particular, it increases the levels of cholesterol and short-chain saturated fatty acids [5]. Furthermore, the identification of adulteration is important for consumers who avoid the consumption of cow milk for allergy or cultural reasons [6]. This practice has prompted the Italian government to reinforce controls on food mislabeling and fraud through traceability mechanisms [7] by means of a specific decree [8], thus guaranteeing the PDO denomination and ensuring strict compliance with the specifications of European regulations [9,10].

Several methods are used to detect the presence of extraneous milk in dairy products. The isoelectric focusing (IEF) of γ-caseins is the official method for detecting the presence of cow milk protein, which has to be less than 1% in buffalo mozzarella cheese, according to the current EC Regulation No. 213/2001 [11]. In addition to the IEF, High-Performance Liquid Chromatography with UV detection (HPLC-UV) and matrix-assisted laser desorption/ionization time-of-flight mass spectrometry are used to identify milk species in dairy products [3]. Current alternative approaches for detecting fraud in dairy products are Polymerase Chain Reaction-based assays, which are able to identify species-specific nucleotide sequences. Since these techniques are influenced by physiological and non-physiological (e.g., mastitis) conditions, as well as by technological processes [12], their results should be combined with those of other analyses [12]. 

Among the different PCR-based methods, the droplet digital Polymerase Chain Reaction (ddPCR) is a direct and sensitive technique for quantifying nucleic acids of interest. Unlike real-time PCR (qPCR), this method yields absolute concentrations without requiring the presence of a standard calibration curve, thus making the process faster, more precise, and reproducible [13]. Furthermore, the ddPCR is used for several applications such as the detection of viruses, bacteria, and parasites involved in different diseases [14,15]. 

The aim of the present study was to develop and assess a ddPCR technique able to detect the DNA of *Bos taurus* in PDO buffalo mozzarella cheese and compare the results with those yielded by qPCR and protein-detection assays such as IEF and HPLC-UV. 

## 2. Materials and Methods

### 2.1. Samples

A total of 33 buffalo mozzarella cheeses—labelled as PDO products—were selected: 20 from self-control analyses carried out by factories and 13 from official monitoring campaigns conducted by the Health Authority of the Campania Region. All samples were analyzed by means of PCR, qPCR, ddPCR, IEF, and HPLC-UV.

In addition, standard mozzarella cheese samples made with 100% bovine or 100% buffalo milk were produced according to Lopparelli [16]. Other standard samples of buffalo mozzarella cheese containing 0.1%, 0.5%, 1%, and 5% bovine milk were provided by the National Reference Centre for Hygiene and Technologies of Water Buffalo Farming and Production in Salerno. The percentage of 1% was selected because it represents the maximum amount of bovine milk that can be added to PDO buffalo mozzarella cheese, according to the current EC Regulation No. 213/2001. We decided to decrease the percentage of bovine milk to test the sensibility of all methods of analysis. 

A further analysis was carried out to investigate the specificity of the proposed approach. Specifically, two buffalo mozzarella cheeses with different characteristics were analyzed by means of ddPCR and IEF. One was a buffalo mozzarella cheese made from a mixture of buffalo and cow whey; the other was made from buffalo whey and calf rennet (Calza Clemente 80/20, Titer 1/20,000), thus resembling a PDO buffalo mozzarella cheese. 

### 2.2. DNA Extraction

In order to compare the performance of two different extraction kits, DNA extraction from each sample was carried out by means of QIAamp DNA Mini Kit (Qiagen, Milano, Italy) and Ion Force Fast (Generon, San Prospero, Modena, Italy). All extractions were carried out according to the manufacturers’ instructions.

### 2.3. PCR

The PCR reaction mix (50 μL) consisted of 100 ng of DNA, 25 μL of HotStar Taq Master mix (Qiagen GmbH, Hiden, Germany), 1 μL of each primer (10 µM), and up to 50 μL of RNAse/DNase-free water. The target gene for the PCR was the D-loop region of mtDNA of both species. The thermal profile consisted of 15 min at 95 °C; then 35 cycles of 30 s at 94 °C, 1 min at 60 °C, and 1 min at 72 °C; then a final elongation step of 5 min at 72 °C. Amplified PCR products were visualized by means of the QIAxcel Advanced System (Qiagen GmbH, Hiden, Germany), which is a fully automated, sensitive, high-resolution system of capillary electrophoresis. The bovine species was confirmed by the presence of a 147 bp fragment obtained by using the following primers: L8249 For, 5′-CAC AAT CCA GAA CTG ACA C-3′, H8357 Rev, and 5′-GTA GGC TTG GGA ATA GTA CGA-3′ [17]. By contrast, the buffalo species was associated with the presence of a 226 bp fragment, obtained with the following primers: Forward-5′- ACT AGATCA CGA GCT TGATCA CCATGC-3′, and Reverse-5′- GTT ATG TGT GAG CAT GGG CTG ATT GGA-3′ [18].

### 2.4. qPCR

This assay was performed using a 96-well plate (Hard-Shell^®^ 96-Well PCR Plates, #hsp9601, 161 Bio-Rad, Hercules, CA, USA.) and the CFX96 thermal cycler real-time System (Bio-Rad), according to the following thermal profile: 50 °C for 2 min, 95 °C for 10 min, 40 cycles of two steps of 95 °C for 15 s, and 60 °C for 1 min [19]. 

The target gene was cytB. The reaction mix (50 μL) consisted of: 1× TaqMan Universal Master Mix (Applied Biosystems, Hilden, Germany), 0.9 µM of each forward and reverse primer with 100% homology between cow and buffalo, 0.1 µM of probe (Table 1), 5 μL of the DNA sample (100 ng), and up to 50 μL of RNAse/DNase-free water [19]. Each sample was analyzed in duplicate and in two independent qPCR assays. Data acquisitions and analyses were performed by means of the Bio-Rad CFX Maestro software (Bio-Rad). The positive controls used for the ddPCR were also used for the qPCR. The results were expressed as copies/µL and were compared with the number of copies/µL detected by the ddPCR in standard samples).

### 2.5. ddPCR

The reaction mix used for the ddPCR (20 µL) consisted of: the ddPCR Supermix for Probes (no dUTP, Bio-rad) at a final concentration of 1× 0.9 μM primer, 0.25 μM of probe (Applied Biosystems, Waltham, MA, USA), and 5 μL of DNA sample (100 ng). The mix was brought up to 20 µL by adding RNAse/DNase-free water. The primers and probes were the same as those used for the qPCR, as was the gene target. The Mastermix and sample DNA were thoroughly mixed and transferred to a DG8 Cartridge for a QX100™ Droplet Generator (Bio-Rad). Next, the Droplet Generation Oil for Probes (Bio-Rad) was added to the cartridges, which were placed into the QX100™ Droplet Generator (Bio-Rad). After droplet generation, 40 µL (total volume of droplets) were carefully transferred to a twin-tec semi-skirted 96-well PCR plate (Eppendorf AG, Hamburg, Germany), which was then heat-sealed. Subsequent amplification was performed by using a T100 Touch thermal cycler (Bio-Rad). The thermal profile consisted of: 95 °C for 10 min (enzyme activation), 40 cycles of two steps of 94 °C for 30 s, 60 °C for 1 min, 1 cycle at 98 °C for 10 min (enzyme deactivation), and finally, holding at 12 °C. The droplets were read by the QX100™ Droplet reader (Bio-Rad), while the ddPCR data analyses were carried out by means of Quantasoft Version 1.4. Furthermore, each reaction was carried out in duplicate and a negative sample was included. The concentration of bovine DNA in buffalo mozzarella cheese was finally expressed as copies/µL.

### 2.6. Specificity and Sensitivity of PCR, qPCR, and ddPCR

The specificity and sensitivity of the PCR, qPCR, and ddPCR was determined by evaluating the reproducibility of the results obtained by analyzing the DNA aliquots extracted from buffalo mozzarella cheese containing different fractions of cow milk (ranging from 0.1% to 5%). Each condition was assessed in duplicate and carried out in two independent experiments or runs.

### 2.7. Isoelectric Focusing (IEF)

IEF was carried out as reported by Capuano [20] in order to confirm the presence of buffalo and/or cow milk in all samples of buffalo mozzarella cheese. Briefly, 2.5 gr of the sample was suspended (1/10 *w*/*v*) in 25 mL of 0.05 M sodium tetraborate buffer at pH 9 (Sigma, Merck KGaA, Munich, Germany). After homogenization, centrifugation (2000× *g* × 5 min), and fat layer elimination, 500 µL of this sample was mixed with 12 µL of bovine plasmin (Boehringer, Ingelheim, Germany) and incubated at 37 °C for one hour. Later, 500 µL of 24% trichloroacetic acid (*w*/*v*) was added to the sample. After centrifugation at 2000× *g* × 5 min, the precipitate was eluted in 200 µL of urea 9 M (Sigma, Merck KGaA, Munich, Germany). An aliquot of 4 µL was used for the subsequent IEF, carried out by means of the PhastSystem (Amersham Bioscience, Uppsala, Sweden) horizontal equipment. The certified c-casein standards from buffalo (Department of Agriculture, University of Naples, Italy), cow, goat, and sheep (Sigma, Merck KGaA, Munich, Germany) were also used as controls in this assay. After electrophoresis, the gel was stained as described by Olsson [21], and the results were evaluated in accordance with the CE Regulation 273/2008. 

### 2.8. High-Performance Liquid Chromatography (HPLC) 

HPLC with UV detection were carried out for the identification and quantification of β-lactoglobulin A (β-LG A) in all buffalo mozzarella cheese samples containing different fractions of cow milk. 

About 4.0 g of minced cheese was centrifuged at 12,000× *g* for 15 min at 4 °C. The supernatant was added, while stirring, to an equal volume of acetic acid/sodium acetate buffer (pH = 4.6). After incubation for 20 min at room temperature, the sample was centrifuged at 2000× *g* for 15 min at 4 °C, filtered through a 0.22 µm membrane (Millipore Corp., Bedford, MA, USA), and finally, analyzed by means of the HPLC method. A blank sample was prepared by replacing the aqueous phase from cheese with distilled water.

The HPLC system was equipped with a quaternary valve (model 1200 Agilent, Santa Clara, CA, USA). The separation was performed on a C3 RP column (250 mm × 4.6 mm) with 300 °A pores and 5 µm-sized particles (Agilent), and the detection wavelength was set at 205 nm. The analysis was carried out by applying a gradient of the mobile phases HPLC-grade water containing 0.1% *v*/*v* TFA (trifluoroacetic acid; Carlo Erba, Milan, Italy) (eluant A), and the HPLC-grade acetonitrile (Merck, Darmstadt, Germany) containing 0.1% *v*/*v* TFA (eluant B). Processing and data acquisition were carried out by means of the Agilent Chemstation software and in accordance with the Decree of 10 April 1996, issued by the Ministry of Agricultural, Food, and Forestry Policies [22].

## 3. Results and Discussion

**Figure 1:**
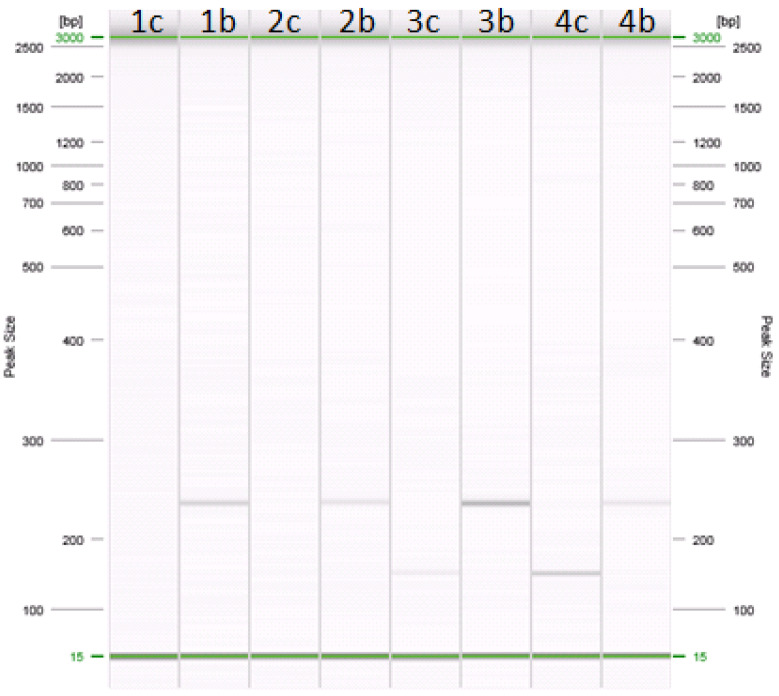
Amplification of cow and buffalo specific primers in PCR. Lanes: (1c–b) 0.1% cow in buffalo mozzarella cheese; (2c–b) 0.5% cow in buffalo mozzarella cheese; (3c–b) 1% cow in buffalo mozzarella cheese; (4c–b) 5% cow in buffalo mozzarella cheese. Samples in lanes 1c, 2c, 3c, and 4c were amplified with primers specific for cow (about 147 bp). Samples in lanes 1b, 2b, 3b, and 4b were amplified with primers specific for buffalo (about 226 bp).

**Figure 2:**
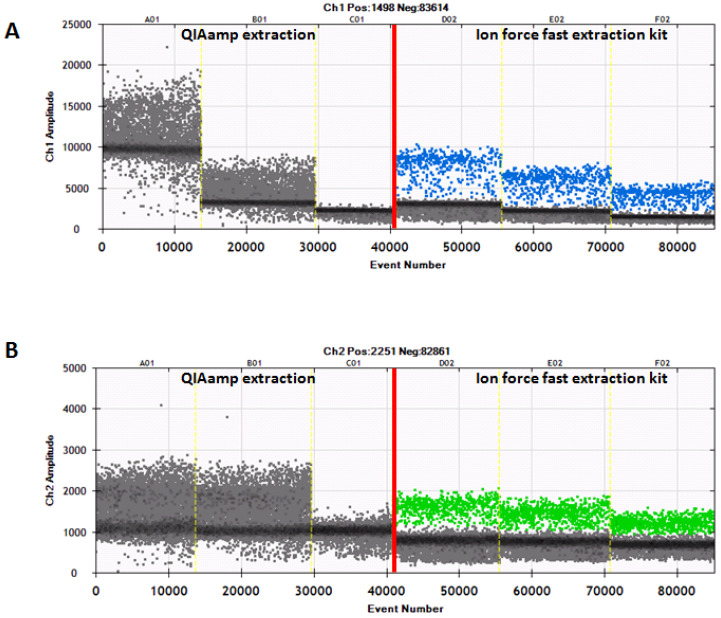
The ddPCR of representative samples extracted by the QIAamp mini kit or the Ion Force Fast kit. (**A**) The detection of bovine DNA is indicated by blue droplets. (**B**) The detection of buffalo DNA is indicated by green droplets.

The gene targets most frequently used to detect different species are *CytB* and *D-loop*; both targets are present in the mtDNA [19,23], which in the case of milk, is found only in the somatic cells [19]. The fact that the number of these cells depends on the physiological and non-physiological (e.g., inflammation) status of the udder and on cheese production technologies can influence the extraction and quantification of DNA from different milk species in foods such as buffalo mozzarella cheese [3], making it difficult to compare the PCR-based assays with the IEF assay. For this reason, two DNA extraction kits and three PCR-based methods (the PCR, the qPCR and the ddPCR) were selected for the extraction of DNA from all samples. The two DNA extraction kits allowed us to obtain a good yield and quality of DNA for the PCR and the qPCR (Figure 1 and Figure 2), while in the case of the ddPCR, the best result (yield and quality of DNA) was obtained by using the DNA extracted by means of the Ion Force Fast kit (Figure 2). In particular, the results of the ddPCR analysis showed a clear distinction between samples containing bovine (blue droplets) and/or buffalo (green droplets) DNA or samples without DNA (grey droplets) (Figure 2A,B). 

Furthermore, the results of the ddPCR showed the threshold of fluorescence probe-Fam (blue droplets) for bovine DNA at an amplitude ranging from 2000 to 3000, while the fluorescence threshold of probe-Vic (green droplets) for buffalo DNA was seen at about 1000. By contrast, in the case of samples extracted with the QIAamp kit, there was no such clear separation; most of the positive droplets appeared negative. The above results allowed us to conclude that the Ion Force Fast kit provides a good yield and quality of DNA, which produced an optimal performance when used for the ddPCR analysis. All the results obtained by analyzing the 33 samples confirmed the presence/absence of cow milk when tested according to the different methods of analysis (the qPCR, the ddPCR, the IEF, and the HPLC-UV) (Table 2), highlighting the specificities of the five methods and the overlap of their results.

The specificity and sensitivity of both nucleic acid-based methods (the PCR, the qPCR, and the ddPCR) were tested on standard samples provided by the National Reference Centre for Hygiene and Technologies of Water Buffalo Farming and Production in Salerno. The results revealed that the ddPCR has greater a sensitivity than of the PCR and the IEF. 

First of all, all tested methods were able to detect the presence/absence of bovine milk in the two standard samples (buffalo mozzarella cheese made with 100% bovine milk or 100% buffalo milk) (data not shown). Furthermore, the qPCR and the ddPCR were both able to detect the presence of 0.1% and 0.5% bovine milk in buffalo mozzarella cheese samples, while the PCR and the IEF were only able to identify bovine milk at values of 1% or 5% (Table 3). The number of initial somatic milk cells was 116,000 for buffalo and 840,000 for cow. 

**Figure 3:**
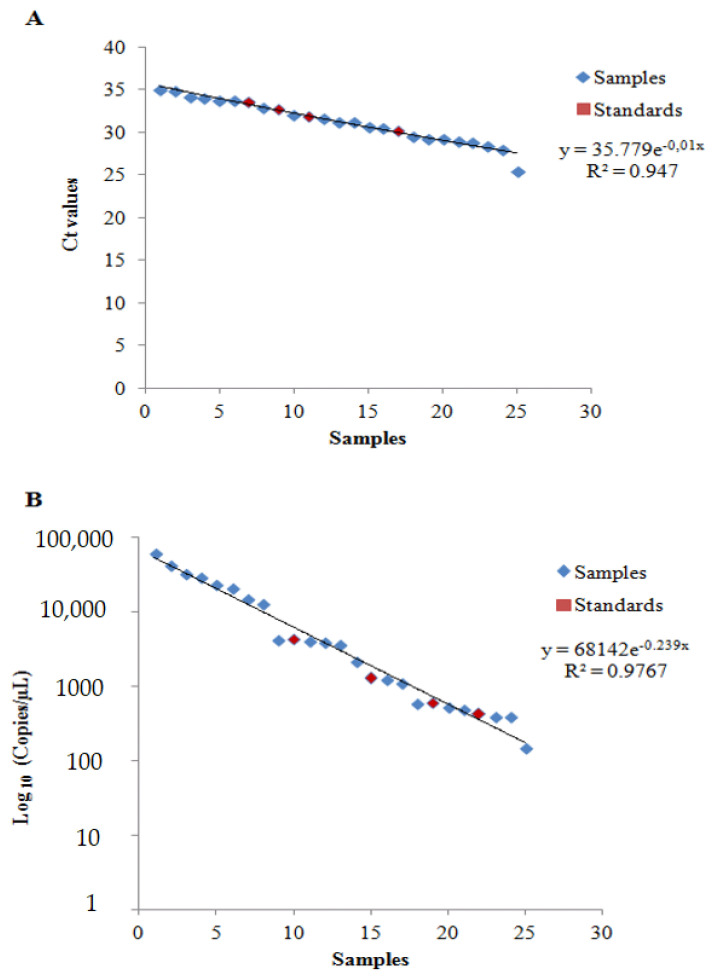
Scatter plot of (**A**) qPCR and (**B**) ddPCR analyses.

On the basis of these data, we also constructed a scatter plot of the results yielded by both PCR-based method assays (Figure 3A,B).

The r-squared values reported in Figure 3 clearly evidenced that both of the proposed models were a good fit with the results yielded by the qPCR (R^2^ = 0.947) and the ddPCR (R^2^ = 0.9767) analyses of the samples and standards.

On the basis of all the results of this research, we can state that the ddPCR is mainly suitable when excellent accuracy of results is required, while the qPCR is preferable if high detection efficiency is needed [24]. This statement is supported by the different SD values between these two methods (Table 3). The lower standard deviations of the ddPCR indicate that the values detected tend to be very close to the mean of the results, while the high standard deviations of the qPCR indicate that the values are spread out over a wider range.

Our additional investigation of the specificity of the proposed approach revealed the ability of the ddPCR to detect bovine DNA in buffalo mozzarella cheese containing mixed buffalo–cow whey and, at the same time, the inadequacy of the IEF in detecting this fraud.

The other mozzarella cheese, which contained only calf rennet, was negative with both methods of analysis (data not shown).

Finally, it is important to point out that the ddPCR is an absolute quantification technique used to calculate the copy number without standard curve and Ct values; it therefore avoids the discrepancy produced by differing amplification efficiencies of the samples. Moreover, this technology is able to detect low quantities of target DNA, even in the presence of inhibitors naturally contained in food matrices. On the other hand, it is also important to highlight that this technique is influenced by physiological and non-physiological (e.g., mastitis) conditions. and by technological processes [12]. Thus, its results should be combined with those of other analyses [12]. Furthermore, Table 4 reports all differences between the individual methods of analysis. 

## 4. Conclusions

Based on our knowledge and on bibliographic studies, this research can be considered the first study focused on the application of the ddPCR for detecting PDO buffalo mozzarella cheese adulteration. In order to demonstrate the efficacy of the proposed approach, we compared it with different methods of analysis such as IEF (the official method for detecting the presence of cow milk) and HPLC-UV, and with other PCR-based assays (PCR and qPCR). This study demonstrated the need to select an appropriate and efficient extraction kit in order to perform a reproducible ddPCR analysis. Indeed, in this study the efficacy of the Ion Force Fast kit allowed us to obtain DNA suitable for ddPCR from the matrix selected (mozzarella cheese). The results indicate that the ddPCR is an effective means of supporting the official controls aimed at combating the adulteration of buffalo mozzarella cheese. Although the ddPCR requires qualified personnel and has comparable costs to those of other methods, such as IEF or qPCR, our results showed that it has greater sensitivity, which makes it an efficient method for detecting fraud, even when small quantities of cow milk are added. At the same time, its high sensitivity makes it difficult to ascertain whether its findings are due to fraud or to accidental contamination during processing. For this reason, it is advisable to use a different manufacturing chain when PDO buffalo mozzarella cheese is produced. Future intra-laboratory tests should be carried out to validate this method for routine tests.

## Figures and Tables

**Table 1:** List of primers and probes used for the ddPCR and qPCR analyses.

**Primers**	**Sequence**
Cytb-forward	5′-AATACACTACACATCCGACACAACAA-3′
Cytb-reverse	5′-GCTCCGTTTGCGTGTATGTATC-3′
**Probes**	
Cow	5′-FAM-CTCTGTTACCCATATCTG-3′
Buffalo	5′-VIC- CCTCCGTCGCCCACA-3′

**Table 2:** ddPCR, qPCR, IEF, and HPLC-UV of buffalo mozzarella cheese samples.

Sample n.	Origin	ddPCR (Copies/µL) ^a^	qPCR (Ct) ^a^	PCR ^a^	IEF ^b^	HPLC-UV ^c^
1	OM	1262.8	34.95	+	+	27
2	SC	492.8	33.82	+	+	19.6
3	OM	15532	28.48	+	+	37
4	SC	4197.6	30.59	+	+	5.6
5	OM	403.48	28.13	+	+	2.7
6	SC	393.36	30.79	+	+	29
7	SC	4408	32.80	+	+	25
8	SC	2217.6	34.23	+	+	11.1
9	SC	44,176.6	31.35	+	+	11.6
10	OM	61,952	25.47	+	+	2
11	SC	21,660	29.32	+	+	13
12	SC	32,920	29.08	+	+	29
13	SC	30,040	28.97	+	+	15.3
14	SC	13,470	30.13	+	+	7.5
15	OM	23,600	29.41	+	+	15
16	SC	546.2	34.11	+	+	17
17	SC	3688	31.35	+	+	11.9
18	OM	1148	35.05	+	+	6.9
19	SC	151.2	33.80	+	+	1.9
20	OM	596.8	31.84	+	+	7.1
21	OM	4000	32.10	+	+	13.5
22	OM	-	-	-	-	-
23	SC	-	-	-	-	-
24	OM	-	-	-	-	-
25	SC	-	-	-	-	-
26	SC	-	-	-	-	-
27	OM	-	-	-	-	-
28	SC	-	-	-	-	-
29	SC	-	-	-	-	-
30	SC	-	-	-	-	-
31	OM	-	-	-	-	-
32	OM	-	-	-	-	-
33	SC	-	-	-	-	-

Note. Values of copies/µL or Ct refer only to cow DNA; (SC): Self-Control analyses; (OM): Official Monitoring campaigns; (ddPCR): droplet digital PCR; (qPCR): quantitative real-time PCR; (IEF): Isoelectric Focusing; (HPLC-UV) High-Performance Liquid Chromatography with UV detection; (+): presence of cow species; (-): absence of cow species; (a): molecular assays targeted on the Cytb gene; (b): presence of cow caseins; (c): % of cow serum proteins.

**Table 3:** Target copies (copies/µL) or Ct detected by ddPCR, qPCR, PCR, and IEF in standard samples.

Standard	ddPCR (Copies/µL)	qPCR (Copies/µL)	PCR	IEF
0.10%	455.5 ± 6.36	398 ± 127.27	-	-
0.50%	602 ± 8.48	447 ± 24.04	-	-
1%	1284.5 ± 21.92	996 ± 121.62	+	+
5%	4355 ± 7.07	3058 ± 93.33	+	+

Note. Values of copies/µL or Ct refer only to cow DNA and are expressed as media ± SD; (+): presence of cow species; (-): absence of cow species.

**Table 4:** Differences between individual methods of analysis.

Method	Type of Response	Sensitivity (%)	Time Analysis (h)	Cost (€)	Implementation Difficulties
PCR	Qualitative	1%	4	20	+/-
IEF	Qualitative	1%	7	30	+
qPCR	Quali-quantitative	0.1%	4	30	+
ddPCR	Quantitative	0.1%	4.30	30	+
HPLC-UV	Quantitative	0.5%	24	56.25	+

Note. (%): Percentages of bovine milk added to PDO buffalo mozzarella cheese; (h): Hours; (€): Euro; (+): High; (+/-): Medium; (-): Low.

## Data Availability

The data presented in this study is contained within the article.
